# Genetic insights into myeloproliferative neoplasms and unusual sites thrombosis

**DOI:** 10.1007/s00277-025-06606-5

**Published:** 2025-09-29

**Authors:** Erika Morsia, Paola Ranalli, Stefano Baldoni, Stefania. Mancini, Sonia Morè, Chiara Cantò, Dorela Lame, Gaetano La Barba, Antonella Poloni, Serena Rupoli, Mauro Di Ianni

**Affiliations:** 1 Department of Clinical and Molecular Sciences (DISCLIMO), Università Politecnica delle Marche, Via Tronto 10/A, Ancona, 60126 Italy; 2 Hematology Clinic, Azienda Ospedaliero Universitaria delle Marche, Ancona, Italy; 3 Department of Medicine and Aging Sciences, University “G. D’Annunzio” of Chieti- Pescara, Pescara, Italy; 4 Hematology Unit, S. Spirito Hospital, Pescara, Italy

**Keywords:** Myeloproliferative neoplasm, Splanchnic vein thrombosis, Cerebral vein thrombosis, Molecular profile

## Abstract

Myeloproliferative neoplasms (MPNs) are associated with an elevated risk of thrombosis in unusual sites such as the splanchnic vein thrombosis (SVT) and cerebral vein thrombosis (CVT). In patients with unusual site thrombosis, screening for MPNs is routine, but diagnosis is often difficult due to the absence of clear clinical signs or elevated blood counts—frequently leading to an MPN-U classification—while the thrombotic event itself is often the first clue. Furthermore, there is no consensus on treatments beyond anticoagulation, leading to variability across centers. This study investigates the molecular characteristics of MPNs associated with SVT and CVT. We conducted a retrospective, multicenter analysis of 44 patients with MPN and unusual site thrombosis from Italian hematology units, using next-generation sequencing. (NGS) to identify driver and passengers mutations. Our findings confirm a high prevalence of unclassifiable MPN (MPN-U) among SVT patients. *JAK2 p.V617F* was found in 86.4% of cases, and patients with additional mutations had higher median *JAK2* variant allele frequencies. *JAK2 p.V617F* is known to promote thrombosis by inducing a pro-inflammatory endothelial environment, particularly relevant in low-flow venous sites such as cerebral sinuses and splanchnic veins, supporting MPN screening in these patients. In contrast, data on *JAK2*-unmutated cases are more limited, but our cohort suggests a possible association between unusual site thrombosis and a more complex mutational profile involving multiple genetic alterations. TET2 mutations were more frequent in patients with MPN-CVT compared to the rest of the cohort (66.6% vs. 15.7%). Absence of *KIT* mutations was associated with poorer thrombotic recurrence-free survival, suggesting a negative prognostic role of *KIT* mutation. Brief report.

## Introduction

Patients with myeloproliferative neoplasms (MPNs) face an increased risk of thrombosis and, to a lesser extent, progression to secondary myelofibrosis or acute myeloid leukemia (AML).

Thrombotic events can occur in unusual sites, such as the splanchnic and cerebral regions [1, 2]. Diagnosing MPN in these individuals is challenging because key indicators, such as elevated blood cell counts and splenomegaly, can be masked by complications like portal hypertension or bleeding. The detection of *JAK2 p.V617F*, *MPL*, and *CALR* driver mutations has been instrumental in improving MPN diagnosis [3]. Subsequent research has shown that testing for these mutations enhances the accuracy of MPN diagnosis in patients with splanchnic vein thrombosis (SVT).

Consequently, this screening with driver mutations should be part of the standard etiological evaluation for patients with SVT. Patients with MPN who present with SVT often exhibit a predilection for younger women and show a strong association with the JAK2V617F mutation. These cases typically have a low JAK2V617F variant allele frequency (generally < 10%) and are characterized by a lower prevalence of CALR, MPL, or JAK2 exon 12 mutations [4, 5]. The use of NGS techniques has further enriched our understanding of the molecular landscape of these cases, offering valuable insights that could enhance both diagnosis and prognosis [6–8]. 

## Materials and methods

We conducted an observational, multicenter, retrospective study on patients with venous thrombosis at unusual sites, specifically cerebral vein thrombosis (CVT) or SVT, who also had a previous or concomitant diagnosis of MPN. The study involved patients treated at the Haematology Units of “Azienda Ospedaliero-.

Universitaria delle Marche” in Ancona and “Santo Spirito” Hospital in Pescara, Italy. A total of forty-four patients with SVT or CVT were selected from a database of patients diagnosed with MPN between 1992 and 2022 at our institutions. *Selected cases showed no predisposing factors for SVT prior to the MPN diagnosis—such as liver cirrhosis*,* other malignancies*,* known thrombophilia*,* or paroxysmal nocturnal hemoglobinuria—all of which were ruled out during follow-up.*

The diagnosis of MPN was made according to the 2008 and 2016 World Health Organization (WHO) diagnostic criteria [9]. All cases of CVT and SVT were confirmed at diagnosis through imaging studies—CVT by brain angiographic computed tomography (CT) and magnetic resonance imaging (MRI), and SVT by abdominal CT scan. Patients were treated in accordance with current recommendations.

Key clinical data at the time of MPN diagnosis, including age, sex, cardiovascular risk factors, complete blood count, LDH, hepatorenal function tests, congenital thrombophilia, complications during the disease course (such as thrombosis, bleeding, progression to myelofibrosis or AML), and status at the last followup were collected. Signed informed consent was obtained from all participants in accordance with local institutional review board requirements.

At diagnosis, DNA samples were collected and initially analyzed by PCR for the detection of driver mutations (*JAK2 p.V617F*,* CALR*, and *MPL*). Subsequently, samples underwent targeted next-generation sequencing (NGS), performed according to the manufacturer’s instructions using the MiSeq platform (Illumina). A custom myeloid gene panel was used, including the following genes: *ABL1*,* ARHGAP31*,* ARID1A*,* ASXL1*,* ASXL2*,* BRAF*,* BRCA1*,* BRCA2*,* CALR*,* CBL*,* CEBPA*,* CSF3R*,* DNMT3A*,* ETV6*,* EZH2*,* FLT3*,* GATA1*,* GATA2*,* HRAS*,* IDH1*,* IDH2*,* IKZF1*,* JAK2*,* JAK3*,* KIT*,* KRAS*,* MLL*,* MPL*,* NF1*,* NOTCH1*,* NRAS*,* NPM1*,* PDGFRA*,* PHF6*,* PIGA*,* PML*,* PTPN11*,* RAD21*,* RB1*,* RUNX1*,* SETBP1*,* SF3B1*,* SH2B3*,* SMC1A*,* SMC3*,* SRSF2*,* STAG2*,* STAT3*,* STAT5B*,* SUZ12*,* TET2*,* TP53*,* U2AF1*,* WT1*, and *ZRSR2*. The study was approved by local institutional review boards.

Qualitative variables were compared through Chi-squared test and quantitative variables through Student’s t-test, Mann-Whitney U test or ANOVA. Overall survival and time-to-event analysis were calculated using the method of Kaplan-Meier with log-rank test for comparisons. Variables evaluated for their potential prognostic significance were age, MPN subtype, and clinical presentation, blood counts at diagnosis, bone marrow fibrosis, driver and additional mutations. Multivariable analyses of the factors predicting the different outcomes were done by Cox regression. All statistical analyses were carried out with R and SPSS software.

## Results

A total of 44 patients were included in the study: PV (*n* = 11), ET (*n* = 12), PMF (*n* = 14), and MPN-U (n.

= 7). CVT accounted for 13.6% of all thromboses (*n* = 6), while SVT represented 86.4% (*n* = 38). Regarding.

SVT localization, 62.1% (*n* = 35) had thrombosis in the spleno-portal area, and 7.8% (*n* = 3) had BuddChiari syndrome. Clinical and hematological features are detailed in Table 1. A higher proportion of patients with MPN-U was observed among those with SVT, consistent with the literature. Only 12.5% of patients exhibited karyotypic abnormalities, with the most common being + 8 and -Y. Although the numbers are very limited, when assessing whether cytogenetic abnormalities were associated with disease characteristics, we found no correlation with clinical phenotype, molecular features (including both driver and passenger mutations), or histopathology. Patients with cytogenetic abnormalities more frequently presented with constitutional symptoms (60% vs. 11.76%, p-value = 0.0208) and were more commonly found among those who had died at last follow-up (33% vs. 6.25%, p-value = 0.0032). No association was observed with thrombotic recurrence.

**Table 1:** Clinical and molecular patients features and outcomes

Characteristics of patients	Tot N = 44	
Age at MPN diagnosis, median (range)	53 (11–80)	
Male, n (%)	19 (43.2)	
MPN subtype, n (%)		
-PV	11 (25)	
-ET	12 (27.2)	
-PMF	14 (31.8)	
-MPN-U	7 (15.9)	
Molecular status, n(%)		**Mean VAF % (range)**
*JAK2 p.V617F*	38 (86.4)	29.6 (1.9–91)
*CALR*	4 (9.1)	47.1 (42.2–51.9)
*MPL*	1 (2.3)	9.0 (9.0–9.0)
Triple-negative	1(2.3)	N.A
NGS, n(%)		**Mean VAF % (range)**
*TET2*	10 (22.7)	41.5 (22.0–48.1)
*KIT*	8 (18.2)	45.3 (42.0–48.0)
*ASXL1*	4 (9.1)	24.8 (5.8–49.1)
*U2AF1*	2 (4.5)	17.6 (8.0–27.3)
*JAK2 non canonic*	2 (4.5)	48.6 (20.2–77.2)
*RUNX1*	3 (6.8)	41.3 (35.3–49.2)
*CSF3R*	2 (4.5)	46.0 (44.4–48.0)
*MYD88*	1 (2.3)	5.1 (5.1–5.1)
*NRAS*	2 (4.5)	9.0 (5.6–12.4)
*GATA2*	3 (6.8)	50.0 (48.0–52.0)
*EZH2*	1 (2.3)	48.1 (48.1–48.1)
*SETBP1*	4 (9.1)	58.5 (43.0–100)
*DNMT3A*	2 (4.5)	10.7 (6.4–15.0)
Other	4 (9.1)	N.A
None	22 (50)	N.A
≥ 3 mutations	15 (34.1)	N.A
Cytogenetic abnormalities, n (%) N tot = 40	5 (12.5)	
Costitutional symptoms, n(%)	8 (18.2)	
Splenomegaly, n (%)	34 (77.2)	
Early fibrosis G 0–1, n (%)	36 (81.8)	
Thrombophilia abnormalities, n (%)	20 (45.5)	
Unusual site thrombosis subtype, n (%)		
SVT	38 (86.3)	
CVT	6 (13.6)	
Thrombosis concomitant with the MPN diagnosis, n (%)	22 (50)	
Follow up from MPN diagnosis months, median (range)	104.15 (15.7–377.8)	
Death, n (%)	9 (20.5)	
Leukemic evolution	2 (4.5)	
Infection	4 (9.1)	
Thrombotic events	3 (6.8)	
Recurrence of thrombosis, n (%)	7 (15.9)	
Median overall survival (C.I 95%)	208.57 (204.02–238.72)	
Median thrombotic recurrence-free survival (C.I. 95%)	NR (288.97-NR)	

*CI* confidence interval, *CVT *cerebral vein thrombosis, *ET* essential thrombocythemia, *MPN* myeloproliferative neoplasms, *MPN-U* unclassifiable MPN, *NA* not applicable, *NR* not reached, *PMF *primary myelofibrosis, *PV *polycythemia vera, *SVT *splanchnic vein thrombosis

Molecularly, 86.4% of patients had the *JAK2 p.V617F* mutation as the driver mutation, with a median *JAK2 p.V617F* variant allele frequency (VAF) of 16.7% (range, 1.89-91.0). In 51.35% of cases, *JAK2* was the sole mutation detected by NGS. Conversely, those with *CALR* or *MPL* mutations showed a much higher incidence of passenger mutations, whereas in 16.7% of these cases, the driver mutation remained the sole genetic alteration identified. Patients with the *JAK2* mutation associated with additional mutations had a higher median *JAK2 P.V617F* VAF compared to patients with the *JAK2* mutation alone in NGS (37.3% vs.

**Fig. 1:**
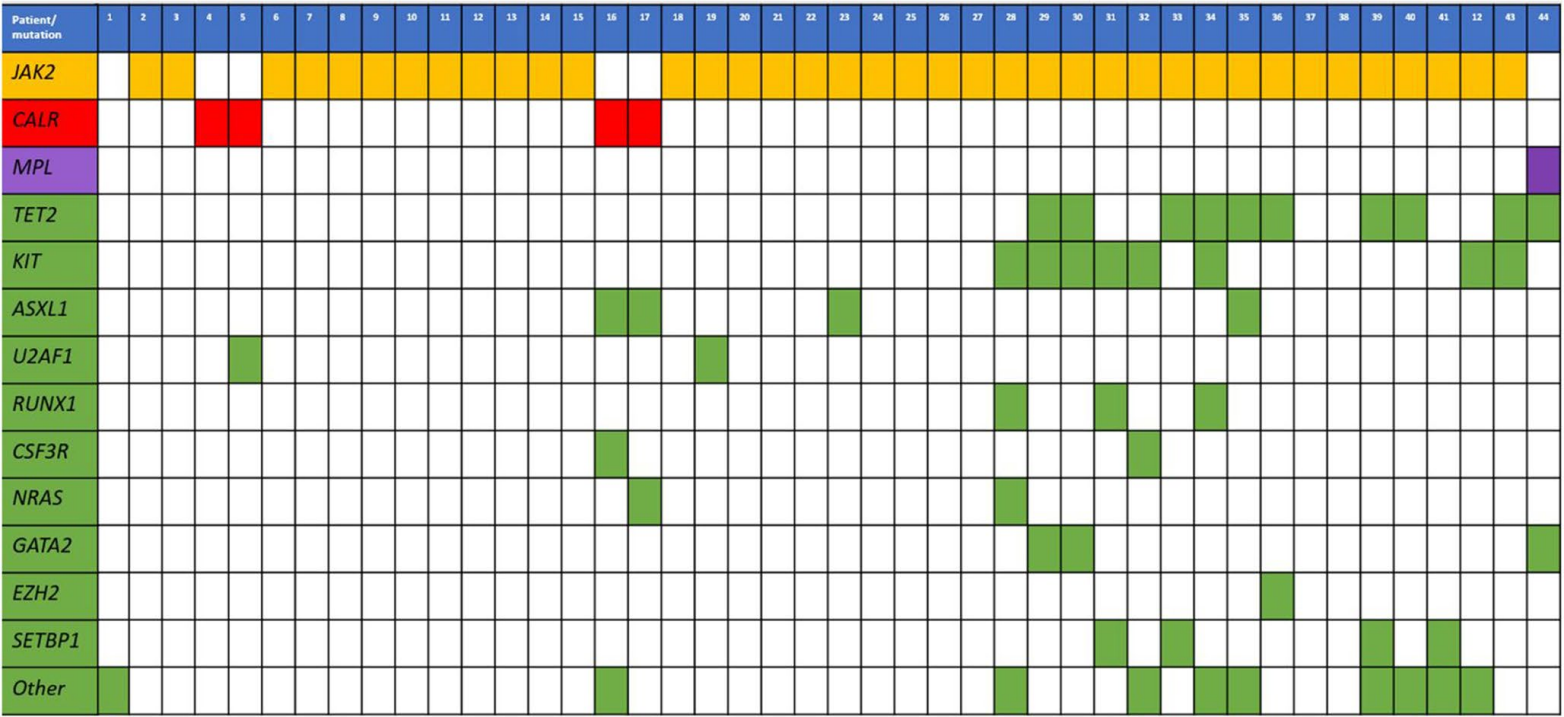
Molecular landscape in 44 patients with MPN associated to unusual site thrombosis

22.3%, pV = 0.06). The mutation landscape is shown in Fig. 1. The main findings in NGS were mutations in *TET2* (22.7%), followed by *KIT* (18.2%), *ASXL1* (9.1%), *SETBP1* (9.1%), *RUNX1* (6.8%), and *GATA2* (6.8%). In 34.1% of cases, three or more mutations were present.

According to the Grinfeld algorithm, no patients presented with the TP53 mutation. In 63.6% of cases, patients fell into the category of MPN with a heterozygous *JAK2* mutation, indicating less molecular complexity, while 25% fell into the category of MPN with chromatin and spliceosome mutations [10]. Comparing clinical and laboratory characteristics between patients with MPN-SVT and MPN-CVT, no differences were observed in terms of sex, type of MPN, or symptoms. A higher proportion of patients with MPN-CVT harbored pathogenic *TET2* variants (66.6% vs. 15.7%; *p* = 0.0057), including frameshift and loss-of-function mutations commonly reported in MPN.

Half of the patients experienced thrombosis at the time of MPN diagnosis. None of them were receiving cytoreductive therapy, although 4 were already on anticoagulant or antiplatelet treatment due to a prior thrombotic event. Among the remaining 22 patients, two were on anticoagulants for previous thrombosis, while 20 were receiving antiplatelet agents for prophylaxis. Cytoreductive therapy was not indicated before thrombosis in 10 patients, as their diagnoses included 5 low-risk PV, 3 low-risk ET, and 2 MPN-U with associated cytopenias. In 2 of these patients, re-evaluation at the time of thrombosis revealed progression to myelofibrosis. Regarding cytoreductive therapy after thrombosis, 61.4% of patients received hydroxyurea, 2.3% received anagrelide, 6.8% received interferon, and 29.5% were treated with JAK inhibitors. In terms of antithrombotic treatment, 56.8% received vitamin K antagonists (VKAs), 15.2% received direct oral anticoagulants (DOACs), and the remaining patients received low molecular weight heparin (LMWH).

With a median follow-up of 8.7 years, 9 patients died. Causes of death are shown in Table 1. In our cohort, age at diagnosis (HR 1.03, 95% CI 1.02–1.05, *p* = 0.00813) and the presence of a *TET2* mutation (HR 1.25, 95% CI 1.18–1.52, *p* = 0.0458) were associated with poor survival in multivariable analysis. Seven patients experienced a new thrombotic event, two of which occurred in unusual sites, with a median thrombotic recurrence-free survival not reached. In multivariable analysis, early stage of fibrosis (HR 1.6, 95% CI 1.3–2.5, *p* = 0.0454), *JAK2 p.V617F* mutation (HR 1.9, 95% CI 1.3–2.7, *p* = 0.0258), and *KIT* wild type (HR 1.4, 95% CI 1.2–2.8, *p* = 0.0288) were associated with worse thrombotic recurrence-free survival.

## Discussion

Herein we describe a series of patients with MPN and unusual site thrombosis: SVT and CVT. This study confirms the high prevalence of unclassifiable myeloproliferative neoplasms (MPN-U) in patients with SVT, highlighting the importance of detailed molecular evaluation in these conditions [11]. Our findings confirmed that *JAK2 p.V617F* mutation alone could play an important role in the development of SVT in MPN patients. In *JAK2* non-mutated patients, the higher frequency of passenger mutations may suggest a potential contribution to the pathogenesis of thrombosis. Furthermore, in our cohort, we observed for the first time a differences in the distribution of the *TET2* mutation, which was more frequently observed in patients with MPN-CVT. Moreover, patients without KIT mutations showed a trend toward reduced thrombotic recurrence-free survival. Mutations such as *ASXL1*,* TET2*,* DNMT3A*,* EZH2*,* RUNX1*, and *U2AF1* are commonly associated with clonal hematopoiesis (CHIP) [12]. However, when detected in patients with MPN, they are typically considered cooperating or passenger mutations. MPNs are characterized by complex clonal evolution, often involving CHIP-related mutations that may precede or coexist with driver mutations, alongside additional passenger lesions. In our cohort, these mutations were considered passenger primarily due to the relatively young age of the patients and the observed VAFs. Although CHIP is mainly observed in older individuals—with a prevalence below 1% in those under 40 and approximately 10% in individuals over 60 years [13]—the median age in our cohort was 53 years, with 65% of patients being under 60. Moreover, while CHIP-related mutations typically exhibit low variant allele frequencies (2–10%), the mutations detected in our series, as shown in Table 1, displayed higher VAFs.

This study has several limitations: although it is multicenter, the sample size is limited and there is no control group, making it impossible to draw definitive conclusions about the association between specific genetic variants and thrombosis at atypical sites. However, it contributes to a better understanding of the molecular heterogeneity in MPN-associated unusual site thrombosis and emphasize the need for large-scale collaborative studies to define the role of the mutational profile and its potential therapeutic implications in this setting.

## Data Availability

No datasets were generated or analysed during the current study.
